# Cost of Contraceptive Implant Removal Services Must Be Considered When Responding to the Growing Demand for Removals

**DOI:** 10.9745/GHSP-D-17-00100

**Published:** 2017-06-27

**Authors:** Jill E Sergison, Randy M Stalter, Rebecca L Callahan, Kate H Rademacher, Markus J Steiner

**Affiliations:** a FHI 360, Durham, NC, USA.

See related article by Christofield and Lacoste.

In a recently published article in *Global Health: Science and Practice*, “Accessible contraceptive implant removal services: an essential element of quality service delivery and scale-up,” Christofield and Lacoste emphasized the growing unmet need for quality implant removal services.^1^ The authors projected the numbers of implant removals in 69 Family Planning 2020 (FP2020) focus countries to more than double between 2015 and 2018 (from 2.2 million to between 4.9 and 5.8 million). The Implant Removal Task Force of the Implants Access Program Operations Group is developing best practices and solutions to meet this increasing demand.

In addition to the sheer numbers of implants that will require removal in coming years, the cost of removal services must also be considered. To begin to understand this impending resource need, we conducted a modeling exercise to forecast potential demand for contraceptive implant removals between 2016 and 2020 in the 5 countries with the highest levels of implant procurement in 2015 (Tanzania, Nigeria, Ethiopia, Kenya, and Zambia).^2^ We then applied a direct cost (supplies and labor) of US$2.41 per removal derived from projections based on previously developed cost estimates for Kenya.^3^

Our analysis differed from Christofield and Lacoste in 2 ways:

1. Instead of assuming all implants are removed at the end of the implants' couple-years of protection (2.5 years to 3.2 years depending on the type of implant), we calculated the number of expected removals per year using cumulative discontinuation rates reported in the most recent Cochrane review assessing the contraceptive effectiveness and acceptability of implants compared with other reversible methods.^4^
2. We estimated the number of implants needed to be removed by 2020 from available Reproductive Health Interchange historical shipment data and from Reproductive Health Supplies Coalition Coordinated Supply Planning procurement projections from 2016–2019.^2^^,^^5^ As with the Christofield and Lacoste model, we assumed a 12-month pipeline delay from in-country receipt of implants to insertion in a client.

According to our modeling, which includes procurement projections beyond 2015, annual implant removal demand rises to approximately 4.5 million by 2020 in just the top 5 implant-procuring countries (Figure). In 2018, our projection for removal demand in these 5 countries is 2.5 million compared with Christofield and Lacoste's projection of 4.9–5.8 million for all 69 FP2020 countries.^1^

**FIGURE:**
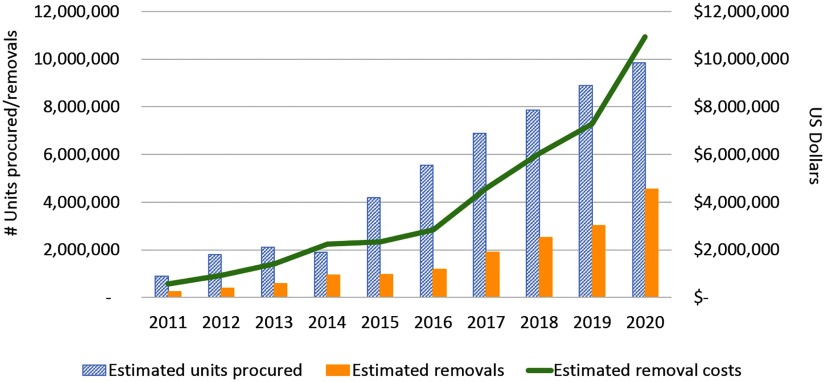
Estimated and Projected Number of Procurements and Removals of Implants, Including Estimated Removal Costs, Among the Top 5 Implant-Procuring Countries^a^ ^a^ Tanzania, Ethiopia, Kenya, Nigeria, and Zambia.

Annual implant removal is projected to rise to about 4.5 million by 2020 in just the top 5 implant-procuring countries.

We estimate the direct cost of providing 4.5 million removals (at $2.41 per removal) will be approximately $10.9 million (about $6.1 million in supply costs and $4.8 million in labor costs). Costs of supplies were taken from both the United Nations Population Fund's (UNFPA's) Access RH Product Catalog (commodity and consumable supplies)^6^ and the IDA Foundation's Electronic Price Indicator (consumable supplies only).^7^ Consumable supplies included sterile gloves, sharps boxes, syringes, scalpel blades, sterile drapes, and anesthetics. Instruments included items such as forceps, bowls, scalpel handles, scissors, and specula.^8^

To calculate a unit cost for reusable supplies, the total cost of the item was divided by an estimated number of procedures. We assumed that nurse-midwives would provide implant insertions and removals,^9^ and labor costs were calculated using labor times from Avenir Heath's OneHealth Tool^10^ and public-sector staff's average salary information in Kenya.^3^ Based on our estimates, more than 22,747 staff hours/week, or 569 full-time equivalents (FTEs), will be required to provide these 4.5 million removals, with nearly 14,500 hours/week (363 FTEs) required in Ethiopia and Tanzania alone (for the more detailed analysis, see the Supplement). Of note, the annual cost estimates are substantially lower when modeling is based on self-reported implant use from Demographic and Health Surveys and Performance Monitoring and Accountability 2020 surveys rather than procurement projections from the Reproductive Health Supplies Coalition.^5^ However, assuming all implants that are procured are eventually used, the total resource demand and removal cost would be the same but would be distributed over additional years.^11^

It will cost approximately $10.9 million to provide the 4.5 million implant removals.

More than 22,000 staff hours/week will be required to provide these 4.5 million implant removals.

The best current source of information about expected demand for removals comes from well-controlled prospective studies. A randomized study of Implanon and Jadelle in 7 countries reported Year 1 and Year 2 continuation rates of 88.2% and 76.3%, respectively.^12^ Similarly, in 2 recent prospective studies of implant use in Kenya, Year 1 continuation rates for implants were 91.8% in one study and 79% in the other.^13^^,^^14^ In other words, one would expect up to 20% of implant users to ask for removals during the first year of use in settings where removal services are readily available.

Some evidence from service delivery data suggests current capacity to provide implant removals may be insufficient even before the expected large increase in demand for removals. A recent synthesis of service statistics in 3 sub-Saharan African countries (the Democratic Republic of the Congo, Tanzania, and Uganda) by EngenderHealth reported 136,737 insertions and only 4,092 removals between January 2014 and June 2016.^15^ Admittedly, these service statistics data have limitations including: (1) timing of the insertions and removals during this 29-month period was not presented, so annual rates cannot be calculated; (2) it is possible that other service delivery groups in these geographic areas where EngenderHealth was providing implant insertions were providing removal services, and thus some removals may not have been captured in the EngenderHealth statistics; and (3) insertion data collection is more complete than removal data collection. Despite these data limitations, the overall 3% removal rate over the 29-month period is sufficiently low to raise questions about adequate access to removal.

A recent synthesis of service statistics data from 3 sub-Saharan African countries reported more than 136,000 implant insertions but only about 4,000 removals between 2014 and 2016.

The need for implant removals will increase dramatically in the coming years, and donors and service delivery programs must be poised to mobilize appropriate resources for quality and timely implant removal services. Dramatic increases in demand for resources for implant removal services will coincide with continued or increasing demand for resources to provide implant insertions. Rigorous collection, synthesis, and analysis of routine service statistics of implant insertions and removals are needed to ensure resources are appropriately balanced in countries such that women have access to high-quality services that guarantee implant removal upon demand.

Donors and service delivery programs must be poised to mobilize appropriate resources for quality and timely implant removal services.

## Supplementary Material

Supplement
