# Supplementary material for: Cost of Contraceptive Implant Removal Services Must Be Considered When Responding to the Growing Demand for Removals
Source: Glob Health Sci Pract. 2017 Jun 27;5(2):330–2. doi: 10.9745/GHSP-D-17-00100 (PMC5487094; doi:10.9745/GHSP-D-17-00100)
Supplement: Supplement [file GHSP-D-17-00100_index.html]

Supplement to Cost of Contraceptive Implant Removal Services Must Be Considered When Responding to the Growing Demand for Removals | Global Health: Science and Practice

## Supplement

Projected implant removals and associated direct costs in top implant procuring countries - 2016-2020 presentation slides

**Files in this Data Supplement:**

- Supplement
