# Supplementary material for: Cost of Contraceptive Implant Removal Services Must Be Considered When Responding to the Growing Demand for Removals
Source: Glob Health Sci Pract. 2017 Jun 27;5(2):330–2. doi: 10.9745/GHSP-D-17-00100 (PMC5487094; doi:10.9745/GHSP-D-17-00100)
Supplement: Supplement [file 17-00100-Sergison-Supplement.pdf]

fhi 360 THE SCIENCE OF IMPROVING LIVES

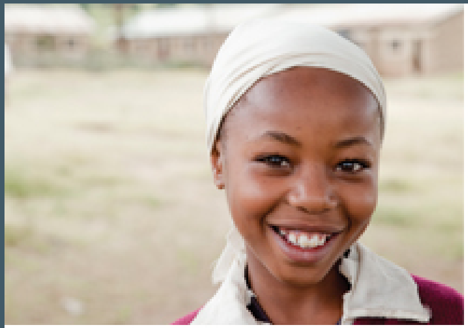

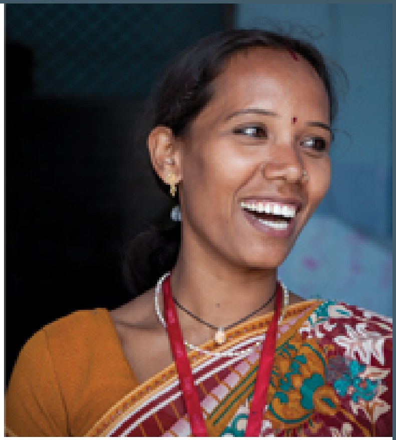

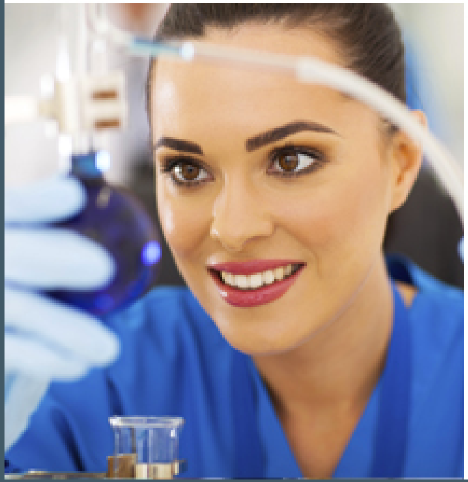

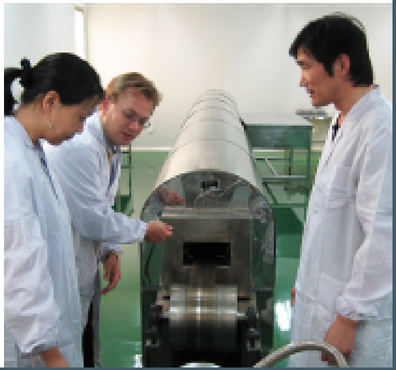

Projected implant removals and associated direct costs in top implant procuring countries - 2016-2020

Randy Stalter, Markus Steiner,
Kate Rademacher and
Rebecca Callahan

March 2017

USAID FROM THE AMERICAN PEOPLE

Rationale

- More than 25 million implants have been purchased for use in less developed countries since 2013
- Evidence has shown that capacity to provide implant removals may be insufficient in many settings
- As need for implant removals increases in coming years, donors and programs should plan to adequately resource this effort

Objectives of Analysis

- To forecast potential demand for contraceptive implant removals from 2016 to 2020 in the countries with the greatest levels of commodity procurement
- To estimate the direct costs (supplies and labor) associated with the projected volumes of removals needed

Methodology

- Focused on five countries with highest 2015 implant procurement figures based on shipment data from RH Interchange database (1)

- Projected removals based on historical and estimated future implant procurement
  - Historical shipment data from years 2011-2015 gathered from the RH Interchange database
  - RHSC Coordinated Supply Planning (CSP) group's implant demand forecast based on blended procurement and use data were used to project post-2015 shipment figures (2)

Methodology (continued)

- Numbers of removals were calculated using cumulative discontinuation rates reported in a 2007 Cochrane review of studies comparing use of Jadelle and Implanon to Norplant (3):
  - Year 1: 8.4%; Year 2: 17.5%; Year 3: 32.6%; Year 4: 82.9%
  - We assumed 100% of implants would be removed by Year 5
- Direct costs (supplies and labor only) of $2.41 per removal were applied to removal projections based on previously developed cost estimates for Kenya (4)

Key Assumptions

- Growth in implant procurement post-2015 for each country would follow global RHSC projections (2)
- A 12-month pipeline delay would exist between in-country receipt of implants and insertion in clients
- Same annual discontinuation rates applied to all implant types
- Costs calculated under assumption that nurse-midwives (or a comparable cadre of health worker) would be performing removals

Key Assumptions (continued)

- Salary information from Kenya is representative of provider remuneration in the other four countries
- Average time of 15 minutes needed to remove an implant, per Avenir Health's (formerly Futures Institute) OneHealth Model (5)
- Work week of 40 hours assumed for calculation of FTEs

Countries Included in Analysis

Countries with greatest 2015 implant shipment numbers*	
Country	*Units shipped*
Tanzania	1,773,427
Ethiopia	1,299,172
Kenya	660,400
Nigeria	564,511
Zambia	376,100

*Based on 2015 RH Interchange shipment data

Results: Tanzania Projections

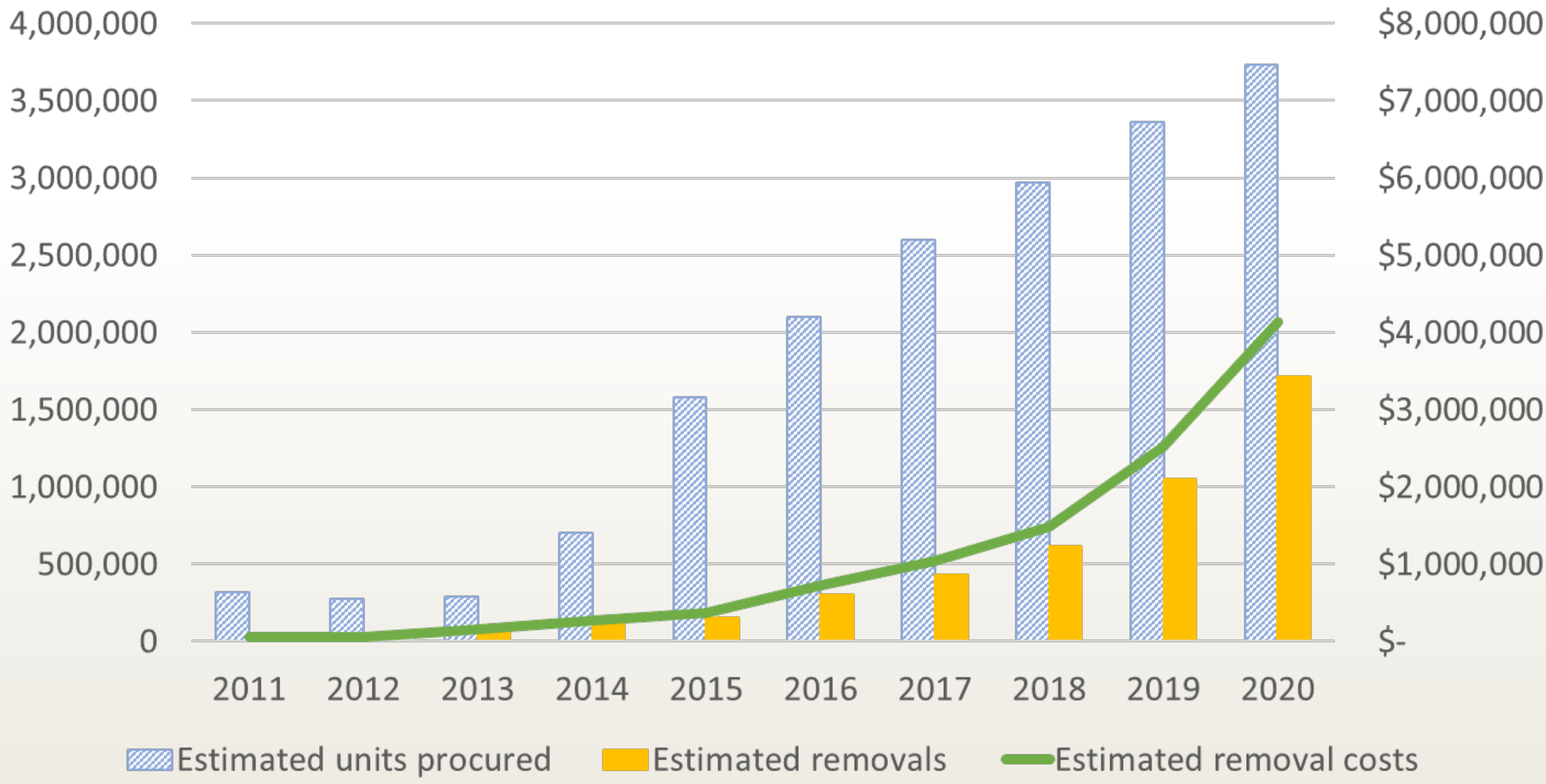

Results: Ethiopia Projections

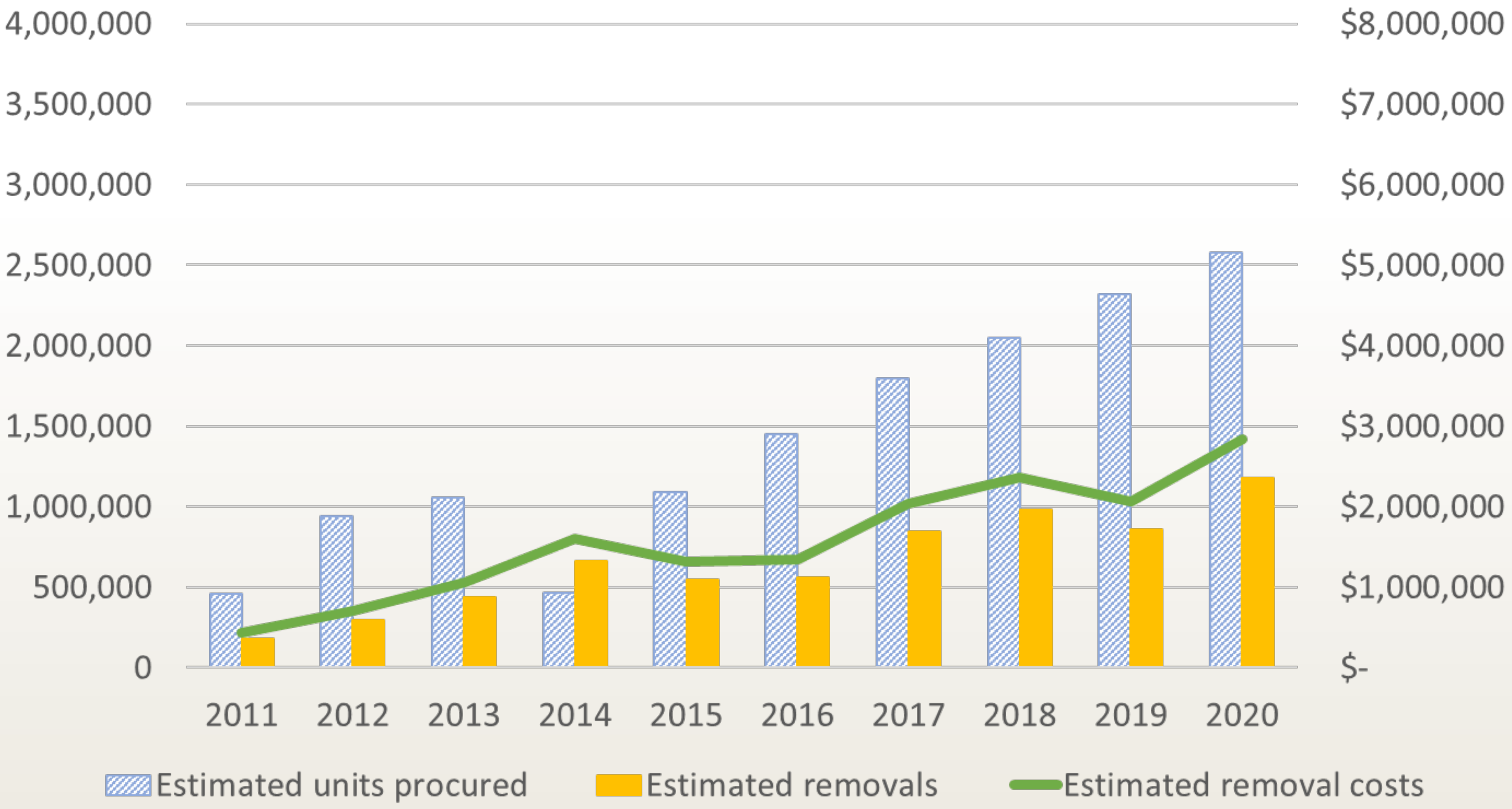

USAID FROM THE AMERICAN PEOPLE

fhi360 THE SCIENCE OF IMPROVING LIVES

Results: Kenya Projections

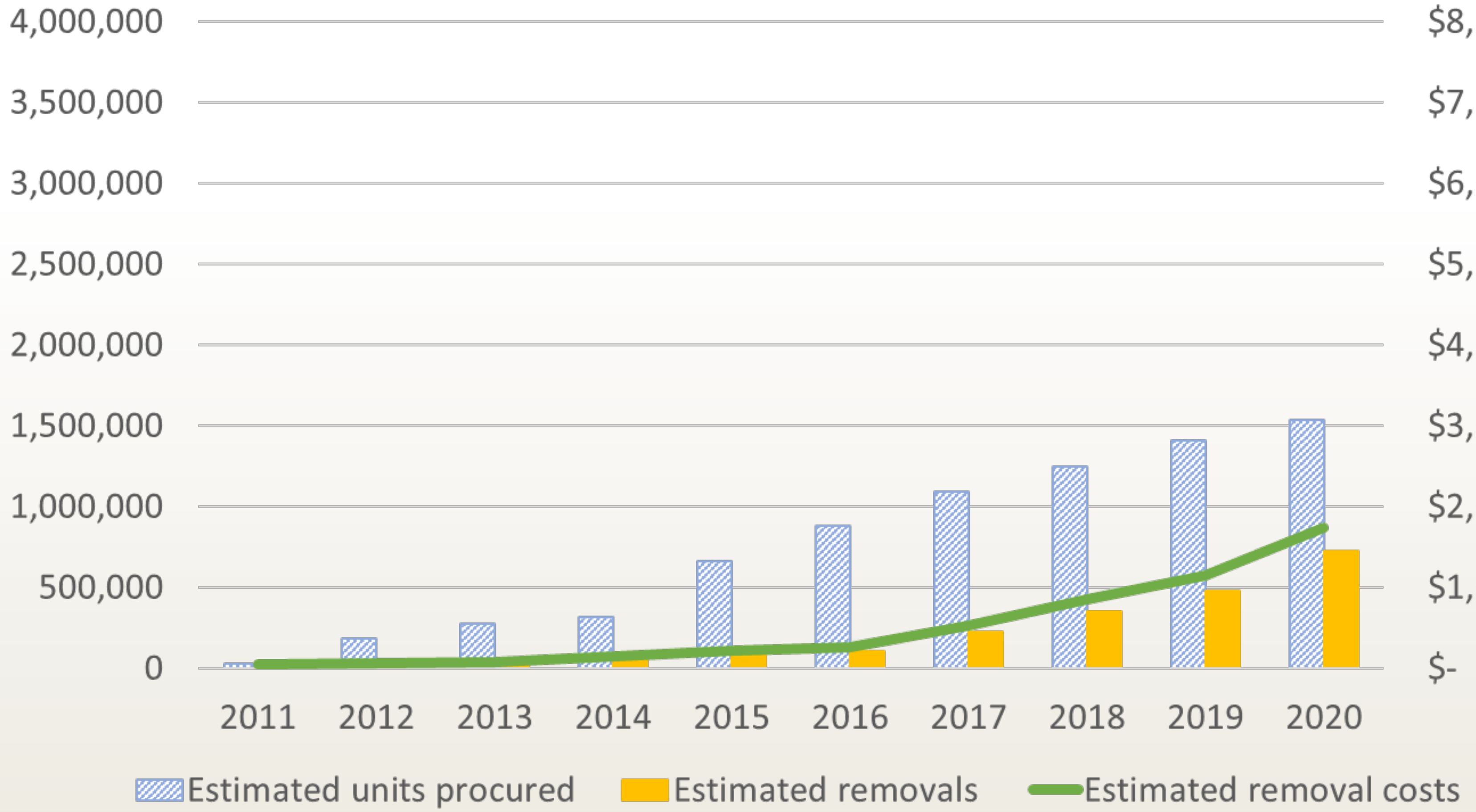

Results: Nigeria Projections

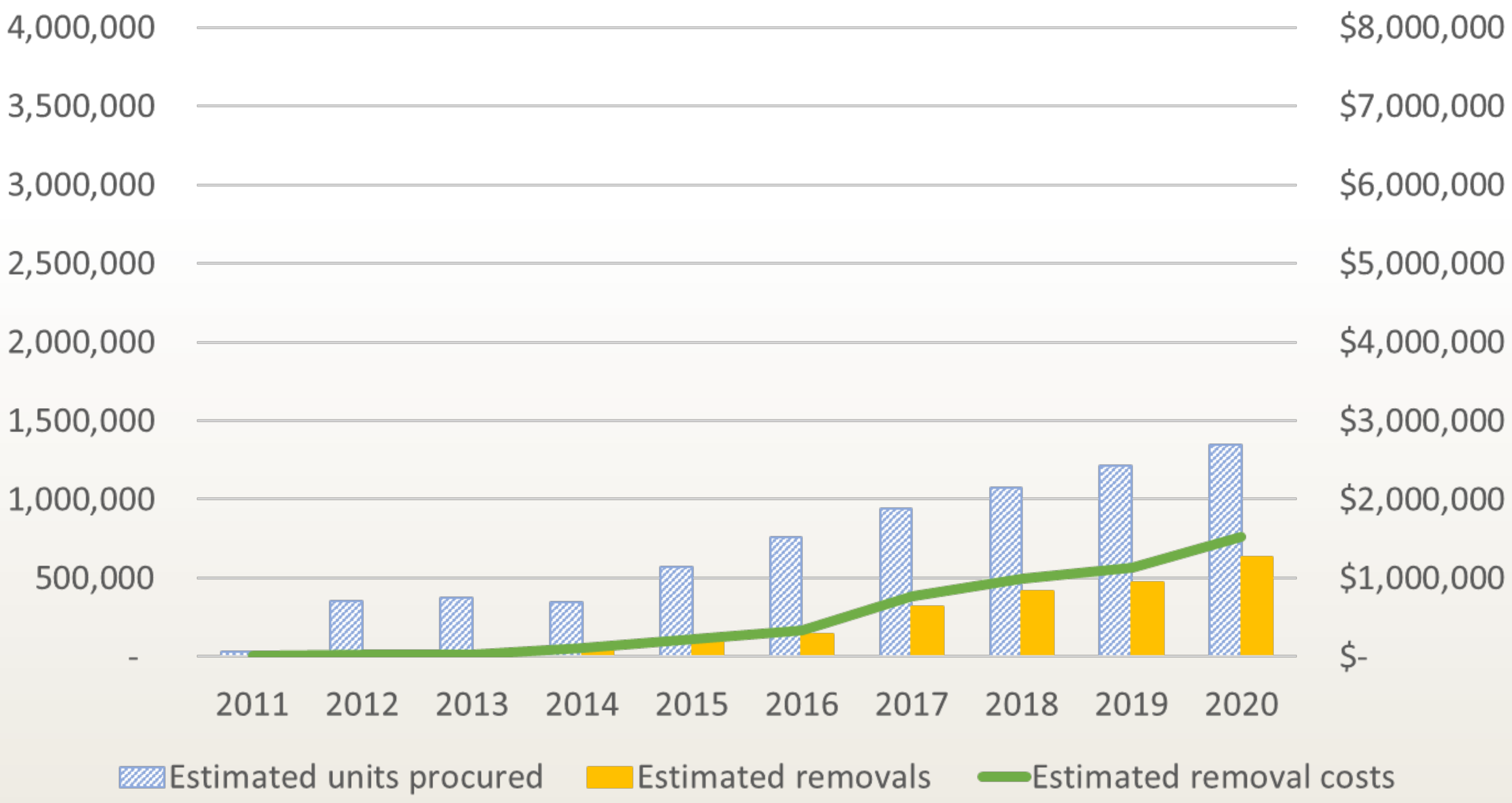

Results: Zambia Projections

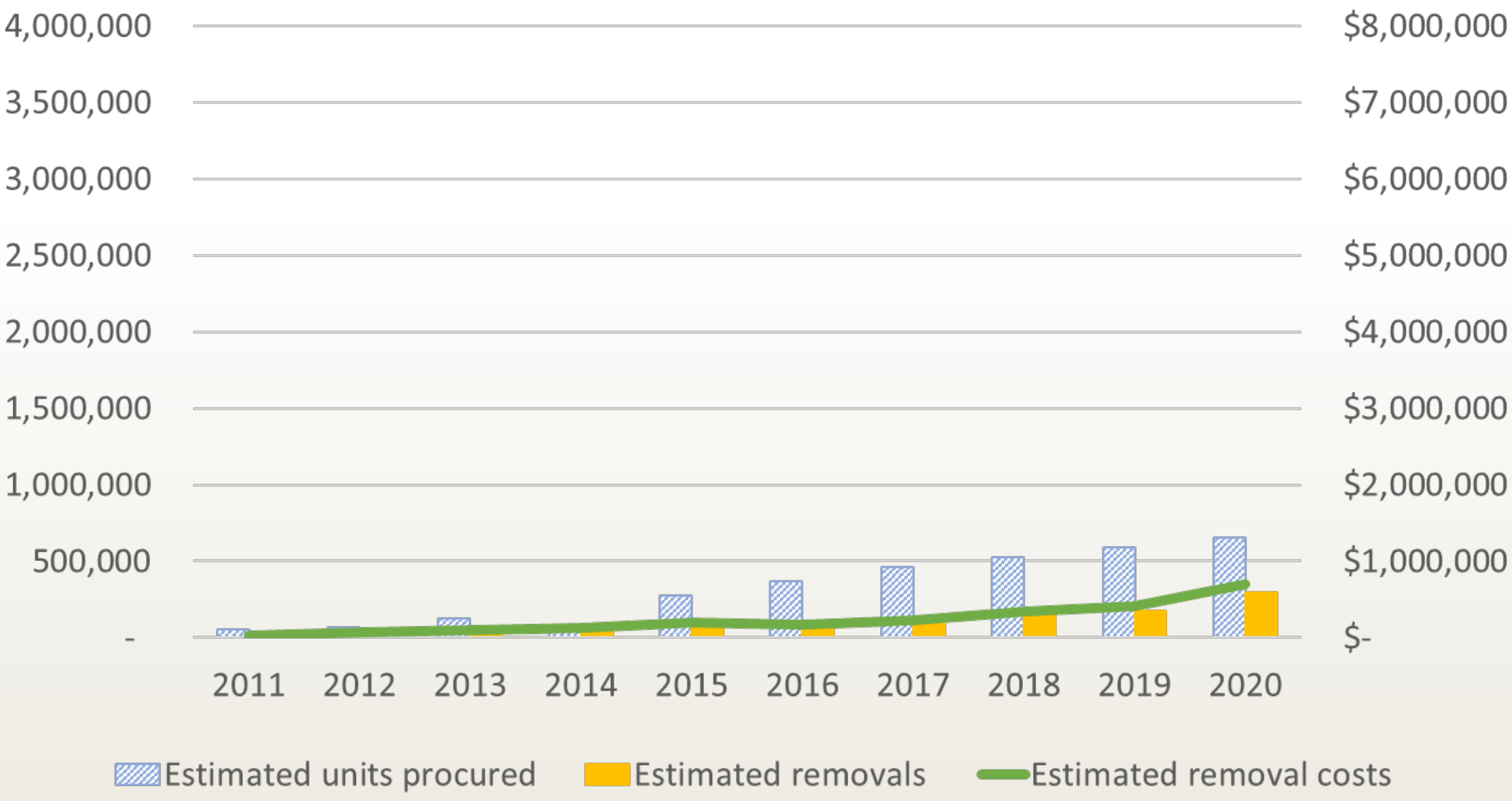

Results: Total projections for all five countries

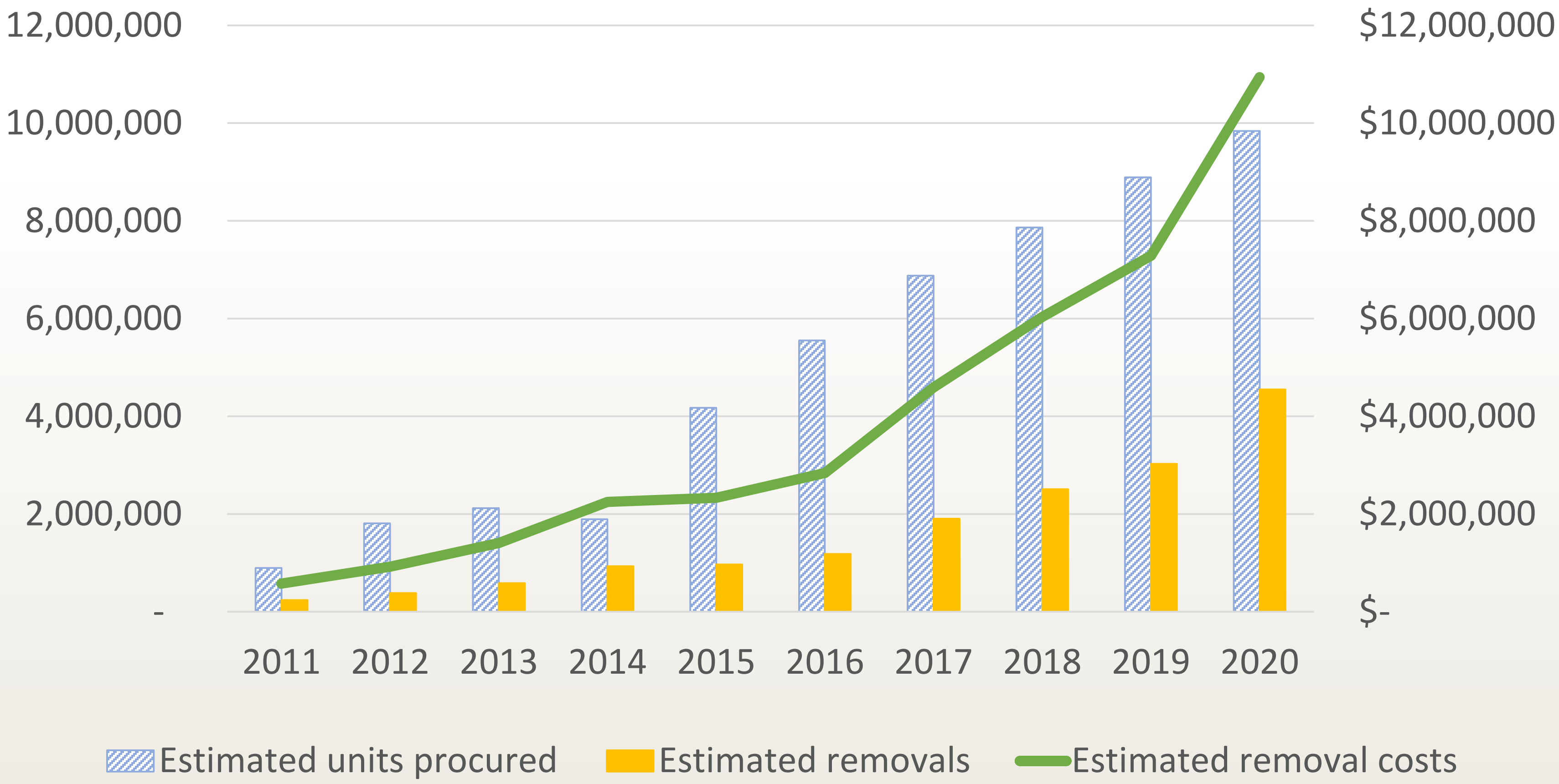

USAID FROM THE AMERICAN PEOPLE
fhi360 THE SCIENCE OF IMPROVING LIVES

Projected removals and associated costs and staffing required in 2020

	Removals	Cost of removals*	Staff hours/week required for removals	FTEs required for removals
**Tanzania**	1,716,388	$4,127,913	8,582	215
**Ethiopia**	1,181,198	$2,840,781	5,906	148
**Kenya**	723,446	$1,739,889	3,617	91
**Nigeria**	634,802	$1,526,698	3,174	80
**Zambia**	293,579	$706,059	1,468	37
**TOTAL**	**4,549,414**	**$10,941,340**	**22,747**	**569**

*Direct costs include costs of supplies and staff time for removal procedure only

Summary of Results

- In the top 5 implant procuring countries, approximately 4.5 million implant removals are estimated to be needed in 2020 according to our procurement-based projections, a 285% increase from 2016 figures

- Direct costs of providing this volume of removals would be about $10.9 million*
  - Approximately $6.1M in supply costs and $4.8M in labor costs

- An estimated 22,747 staff hours per week, or 569 FTEs, would be required to provide all of the necessary removal procedures
  - Nearly 14,500 hours/week (363 FTEs) would be required in Tanzania and Ethiopia alone

- These estimates are substantially lower when the model is based on implant use data (from DHS and PMA 2020 surveys). However, assuming that all implants that are procured are eventually used, total removal costs would be the same in this scenario but distributed over additional years

*Note: In some countries, excess capacity in the health system may be able to absorb some demand for removals; thus, our total cost estimates may overstate actual resource needs. That said, direct costs and staff time consider only the removal procedure; additional resources may be needed for other related activities such as staff training, patient counselling, provision of additional FP methods after implant removal, etc. Further country-specific analyses should be conducted to identify all resource needs within each health system.

Conclusions

- In the context of increasing implant provision, mobilization of resources for removals is crucial
- Number of removals required, as well as cost of removal services, will vary substantially depending on:
  - future procurement levels
  - uptake and continuation rates
  - variation in service delivery models across countries
- A coordinated effort on the part of providers, governments, technical assistance organizations and donors is needed to ensure that quality, affordable removal services are widely available

Data Sources

1. Reproductive Health Supplies Coalition. RH Interchange (RHI) Database. https://www.unfpaprocurement.org/rhi-home. First accessed March 9, 2016.
2. Reproductive Health Supplies Coalition. Summary of April 2016 CSP Contraceptive Implant Demand Forecast. Brussels, Belgium: RHSC, 2016. http://www.rhsupplies.org/uploads/tx_rhscpublications/Summary_of_August_2016_CSP_Contraceptive_Implant_Demand_Forecast.pdf
3. Cochrane Database Syst Rev. 2007 Jul 18;(3):CD001326.
4. Glob Health Sci Pract. 2016 Aug 18;4 Suppl 2:S83-93. doi: 10.9745/GHSP-D-15-00327.
5. Futures Institute. OneHealth model: intervention Treatment Assumptions. Glastonbury, CT: Futures Insitute; 2013. http://avenirhealth.org/Download/Spectrum/Manuals/Intervention%20Assumptions%202013%209%2028.pdf
